# Supplementary material for: Causal inference in multi-cohort studies using the target trial framework to identify and minimize sources of bias
Source: Am J Epidemiol. 2024 Oct 23;194(9):2685–97. doi: 10.1093/aje/kwae405 (PMC12409135; doi:10.1093/aje/kwae405)
Supplement: Web_Material_kwae405 [file web_material_kwae405.zip › AJE-01018-2022_supp material.docx]

**SUPPLEMENTARY MATERIAL**

**Causal inference in multi-cohort studies using the target trial framework to identify and minimize sources of bias**

Marnie Downes, Meredith O’Connor, Craig A Olsson, David Burgner, Sharon Goldfeld, Elizabeth A Spry, George Patton, Margarita Moreno-Betancur

**Table of Contents**

| Appendix S1 |  | Page 2 |
| --- | --- | --- |
| A second case study |  |  |
|  |  |  |
| Table S1 |  | Page 4 |
| Proposed target trial and emulation strategies implicit in the statistical analysis approach of O’Connor et al. (1) for considering the causal effect of exposure to adversity on inflammation in mid- and late-childhood. |  |  |
|  |  |  |
| References |  | Page 8 |

**Appendix S1: A second case study**

**Description of study**

O’Connor *et al.* (1) aimed to investigate the extent to which exposure to adversity negatively impacts inflammation in mid to late childhood, where inflammation was proposed as a central mechanism through which exposure to childhood adversity translates to disease risk, in particular cardiovascular disease risk (2, 3).

*Data sources*

Data from two Australian prospective longitudinal cohort studies were utilized.

The Barwon Infant Study (BIS) is a population-derived birth cohort study (*N*=1074 infants) with antenatal recruitment (at approximately 15 weeks of pregnancy) during 2010–2013, conducted in the Barwon region of Victoria, Australia (4). The study was originally designed to explore the early life origins of a range of non-communicable diseases in the modern environment. Participants completed self-reported structured questionnaires as well as clinical and biological measurements at birth and at 1, 6, 9 and 12 months, and at 2 and 4 years, with the most recent primary school (8–10 years) review commencing in 2020–21. Data on inflammatory biomarkers were available for *N* =510 children at the four-year review. Ethical approval for this methodology was obtained from the Barwon Health Human Research Ethics Committee.

The Longitudinal Study of Australian Children (LSAC) is a nationally representative study of two cohorts, including a birth cohort (*N*=5107 infants), aiming to investigate a broad range of aspects of development and wellbeing over the lifecourse, with 9 waves of bi-annual data collection so far, and wave 10 currently underway. In 2003–2004, a multistage cluster sampling design utilising the comprehensive national Medicare database was employed to select a sample that was broadly representative of all Australian children except those living in remote geographic areas (5). In 2015, a comprehensive, one-off physical health and biomarker module, known as the Child Health CheckPoint, was conducted for the birth cohort between waves 6 and 7, when children were 11–12 years of age (6). Approximately half (53%, *N*=1874 families) of the Wave 6 sample participated in the Child Health CheckPoint (7). The study is overseen by the Australian Institute of Family Studies human ethics review board.

*Objectives of multi-cohort design & published findings*

This study aimed to investigate heterogeneity in the causal effect of exposure to adversity on inflammation across the different outcome measurement time-points of mid-childhood (4 years) and late-childhood (11–12 years), for which replication of analyses were performed. Cohort-specific effect estimates were reported and small associations between exposure to adversity and increased inflammation were consistently observed across both cohorts, however, effects were imprecisely estimated.

**Application of the target trial framework**

Supplementary Table 1 outlines a proposed target trial and corresponding emulation strategies for the two cohorts (BIS and LSAC) implicit in the statistical analysis approach described in O’Connor *et al.* (1). The final column of the table identifies potential remaining “within-cohort biases” not addressed within the analysis approach. Note the specific difference in the causal effect definition for which effect heterogeneity is examined, namely the different outcome measurement time-points, is explicitly defined in the follow-up period protocol component of the target trial.

The paper examined multiple definitions of adversity including a binary exposure to each of several different types of adversity, a cumulative count of the types of adversities experienced, and initial timing of exposure to adversity. For simplicity, here we consider a binary indicator of exposure to any type of adversity.

Given this case study focuses primarily on replication of analyses, we consider possible explanations for discrepant findings across cohorts. These could be attributed to discrepant remaining within-cohort biases, detailed in the final column of Table 1, chance, or alternatively, may be explained by an actual difference in the causal effect across the two time points at which the inflammation outcome was captured by the studies (mid-childhood at 4 years in BIS vs. late-childhood at 11–12 years in LSAC).

**Table S1:** Proposed target trial and emulation strategies implicit in the statistical analysis approach of O’Connor *et al*. (1) for considering the causal effect of exposure to adversity on inflammation in mid- and late-childhood.

| **Protocol component** |  | **Emulation strategies** | | | **Potential remaining**  **within-cohort bias risks** |
| --- | --- | --- | --- | --- | --- |
|  | **Target trial** | **BIS** | **LSAC** | |  |
| 1. ***Eligibility criteria*** | **Target population:**  Australian infants at birth in early 2000s | **Analytic sample selection:**  BIS participants, who were recruited through pregnant women attending antenatal appointments at approximately 15 weeks during 2010–2013, in Barwon region of Victoria (south-east Australia).  **Approach to handling missing data and other potential sources of selection bias:**  All BIS participants were retained in the sample regardless of missing data via use of multiple imputation. | **Analytic sample selection:**  LSAC participants who subsequently participated in the Child Health CheckPoint, a one-off physical health assessment at 11–12 years. LSAC is a cohort of Australian infants aged 0-1 years in 2004 recruited through multi-stage cluster sampling of the comprehensive Medicare database.  **Approach to handling missing data and other potential sources of selection bias:**  All LSAC CheckPoint participants were retained in the sample regardless of missing data via use of multiple imputation. | | - Risk of selection bias due to each study’s sample selection strategy (e.g., calendar period, geographic location, recruitment procedure) capturing only a subset of the target population (e.g., those able to speak and understand English). - Risk of selection bias due to non-participation:   - In BIS, baseline cohort characteristics similar to the Australian population, except a smaller proportion of families from non-English speaking backgrounds.   - In LSAC, baseline cohort characteristics broadly representative of the Australian population, except a smaller proportion of children living in highly remote geographic areas.   - In LSAC, participation in Child Health CheckPoint for outcome assessment required presentation at a testing site for venous blood collection; sample was more socially advantaged than the original cohort. - Risk of selection bias (in each of BIS and LSAC) due to loss to follow-up/missing data in any analysis variable; mitigated in both cohorts by use of multiple imputation on all missing data. |
| 1. ***Treatment strategies*** | **Treatment arms in the trial:**  Intervention arm:  Experience of adversity during childhood  Comparator arm:  No experience of adversity during childhood | **Treatment/Exposure measure:**  Intervention arm:  Exposed to adversity at any measured time point(s) during childhood  Comparator arm:  Never exposed to adversity during childhood  Adversity measured as parent-reported presence of any of seven adverse experiences:   - - Parent legal problems   - Parent mental illness   - Parent substance abuse   - Anger in parenting responses   - Separation/divorce   - Unsafe neighbourhood   - Family member death   Each adversity measured at least once across the waves (but not at all waves):   - - W1 (1 month)   - W2 (6 months)   - W3 (12 months)   - W4 (2 years)   - W5 (4 years) | **Treatment/Exposure measure:**  Intervention arm:  Exposed to adversity at any measured time point(s) during childhood  Comparator arm:  Never exposed to adversity during childhood  Adversity measured as parent-reported presence of any of seven adverse experiences:   - - Parent legal problems   - Parent mental illness   - Parent substance abuse   - Anger in parenting responses   - Separation/divorce   - Unsafe neighbourhood   - Family member death   Each adversity measured at each wave:   - - W1 (0–1 years)   - W2 (2–3 years)   - W3 (4–5 years)   - W4 (6–7 years)   - W5 (8–9 years)   - W6 (10–11 years) | | - Measurement issues (beyond an imprecisely defined intervention):   - The use of imperfect measures of childhood adversity, e.g., parental mental illness measured in BIS using the Edinburgh Postnatal Depression score>13 (depression likely) and in LSAC using K-6 scale>13 (high psychological distress).   - The full range of adversity experienced during childhood not being adequately captured, e.g., racial discrimination.   - Some adversities measured using proxies, for example, anger in parental responses scale used as a proxy for child maltreatment.   - Family circumstances and experience of adversity may alter reporting.   - Adversity indicators sometimes not including the full interval between waves, for example, responses were made in reference to the past 12 months even if waves were >12 months apart, meaning some adverse experiences may not have been captured.   - In LSAC, a change in scale of measurement for anger in parental responses (harsh parenting) between waves 2 and 3.   - In BIS, adversity indicators of unsafe neighbourhood and anger in parenting responses measured at only one wave. |
| 1. ***Assignment procedures*** | **Randomisation strategy:**  Randomisation at recruitment (birth) without blind assignment | **Selection of confounders:**  Confounder (self-reported measure)   - Child sex - Family socioeconomic position (composite of education and income, dichotomised bottom third vs. higher) - Young maternal age (below or above 23 years) - Indoor smoking (Y/N, same room as baby) - Ethnicity (Anglo/European, Ethnic minority) - BMI (continuous) at 4–5 years   **Approach to confounding adjustment:** Outcome regression | **Selection of confounders:**  Confounder (self-reported measure)   - Child sex - Family socioeconomic position (composite of education, occupation and income, dichotomised bottom third vs. higher) - Young maternal age (below or above 23 years) - Indoor smoking (Y/N, any indoor smoking) - Ethnicity (Anglo/European, Ethnic minority) - BMI (continuous) at 4–5 years   **Approach to confounding adjustment:** Outcome regression | | - Risk of residual confounding bias due to unmeasured confounding. - Risk of residual confounding bias due to measurement error:   - The use of proxies for confounders in the absence of more direct measures, e.g., a composite variable of education, occupation and income for socioeconomic position, a composite of language and country of birth for ethnicity, indoor smoking measured using Y/N same room as baby in BIS vs. Y/N any indoor smoking in LSAC.   - Inaccurate reporting of confounders that reveal sensitive information such as income, smoking history. |
| 1. ***Follow-up period*** | **Start and end times:**  Start: At birth  *Difference of interest in effect heterogeneity analysis:*  Endpoint 1: Mid-childhood (4 years)  Endpoint 2: Late-childhood (11–12 years) | **Timing of measures:**  Start: Wave 0, pregnancy  Ends: Wave 5, 4 years | **Timing of measures:**  Start: Wave 1, 0–1 years  Ends: Wave 6.5 (Child Health CheckPoint),  11–12 years | | - Measurement issues due to the exposure not being measured continuously over all childhood nor in the same way for all participants. - Risk of measurement bias due to the outcome not being measured at exactly the specified endpoint (4 years for BIS, 11–12 years for LSAC).   Note: The difference in the outcome measurement endpoints is the key factor of interest in the research question, therefore it is not a bias per se, but the source of difference to be assessed. |
| 1. ***Outcome*** | **Outcome measure:**  Inflammatory markers (continuous, µg/ml):   - - hsCRP   - GlycA | **Outcome measure:**  Inflammatory markers (continuous, µg/ml):   - - hsCRP   - GlycA | **Outcome measure:**  Inflammatory markers (continuous, µg/ml):   - - hsCRP   - GlycA | |  |
| 1. ***Causal contrasts of interest and causal effect measure*** | Percentage difference in mean inflammatory marker levels between intervention and comparator arms in the target population |  | |  | |

**References**

1. O'Connor M, Ponsonby A-L, Collier F, et al. Exposure to adversity and inflammatory outcomes in mid and late childhood. *Brain, Behavior, & Immunity-Health* 2020:100146.

2. Boyce WT, Sokolowski MB, Robinson GE. Toward a new biology of social adversity. *Proceedings of the National Academy of Sciences* 2012;109(Supplement 2):17143-8.

3. Miller G, Chen E, Cole SW. Health psychology: Developing biologically plausible models linking the social world and physical health. *Annual review of psychology* 2009;60:501-24.

4. Vuillermin P, Saffery R, Allen KJ, et al. Cohort profile: the Barwon infant study. *International journal of epidemiology* 2015;44(4):1148-60.

5. Soloff C, Lawrence D, Johnstone R. *LSAC Technical paper No. 1. Sample design*. Melbourne, Australia: Australian Institute of Family Studies; 2005.

6. Clifford S, Davies S, Gillespie A, et al. Longitudinal Study of Australian Children's Child Health CheckPoint Data User Guide. Melbourne: Murdoch Children's Research Institute, 2020.

7. Clifford S, Davies S, Wake M, et al. Child Health CheckPoint: cohort summary and methodology of a physical health and biospecimen module for the Longitudinal Study of Australian Children. *BMJ Open* 2019;9:3–22.
